# Crystal structure of bis­(1,3-bis­{[(1*H*-pyrrol-2-yl)methyl­idene]amino-κ*N*}propan-2-olato-κ*O*)manganese(III) nitrate methanol monosolvate

**DOI:** 10.1107/S1600536814020406

**Published:** 2014-09-17

**Authors:** Seoung Hyun Ahn, Jong Won Shin, Dohyun Moon

**Affiliations:** a Department of Chemistry, Kyungpook National University, Daegu 702-701, Republic of Korea; b Beamline Department, Pohang Accelerator Laboratory/POSTECH 80, Pohang 790-784, South Korea

**Keywords:** crystal structure, propan-2-olate ligand, Jahn–Teller distortion, manganese(III) complex, hydrogen bonding, synchrotron study

## Abstract

The asymmetric unit of the title compound, [Mn(C_13_H_15_N_4_O)_2_]NO_3_·CH_3_OH, contains two independent complex cations, in each of which the Mn^III^ ion is located on an inversion centre. The Mn^III^ ion is coordinated by four N and two O atoms from two 1,3-bis­{[(1*H-*pyrrol-2-yl)methyl­idene]amino}­propan-2-olate ligands, resulting in a distorted octa­hedral geometry. The average Mn—ligand bond lengths in the two complex mol­ecules are 2.074 and 2.079 Å. In the crystal, inter­molecular N—H⋯O hydrogen bonds between the pyrrole group of the ligand and the non-coordinating nitrate ion give rise to a chain structure along [10-1]. The methanol solvent mol­ecule and the nitrate ion are connected by an O—H⋯O hydrogen bond.

## Abstract 2

The Mn^III^ ion in the title compound shows a slightly distorted octa­hedral coordination geometry with two O and four N atoms of the pyrrolyl derivative ligand. In the crystal, inter­molecular N—H⋯O hydrogen bonds between the pyrrole group of the ligand and the uncoordinated nitrate ion give a chain structure along [10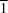].

## Chemical context   

Pyrrolyl derivatives ligands have attracted considerable attention in chemistry and materials science because they can easily be used for the preparation of multifunctional metal complexes with various transition metal ions. These complexes have potential applications in catalysis, and as luminescent materials (Goff & Cosnier, 2011 ▶). For example, a Cr^I,III^ complex with a 2,5-di­methyl­pyrrole ligand has been investigated as a potential ethyl­ene trimerization catalyst (Yang *et al.*, 2014 ▶). Furthermore, zinc complexes containing various pyrrolyl substituents exhibit excellent luminescence properties due to the *n*–π* transitions in the electronic spectra of the pyrrolyl ligand precursors (Gomes *et al.*, 2009 ▶). Here, we report the synthesis and the crystal structure of an Mn^III^ complex with the metal octahedrally coordinated by two anions of 1,3-bis­{[(1*H-*pyrrol-2-yl)methyl­idene]amino}­prop­an-2-ol (Hbpmap), the title compound [Mn(bpmap)_2_]NO_3_·CH_3_OH.
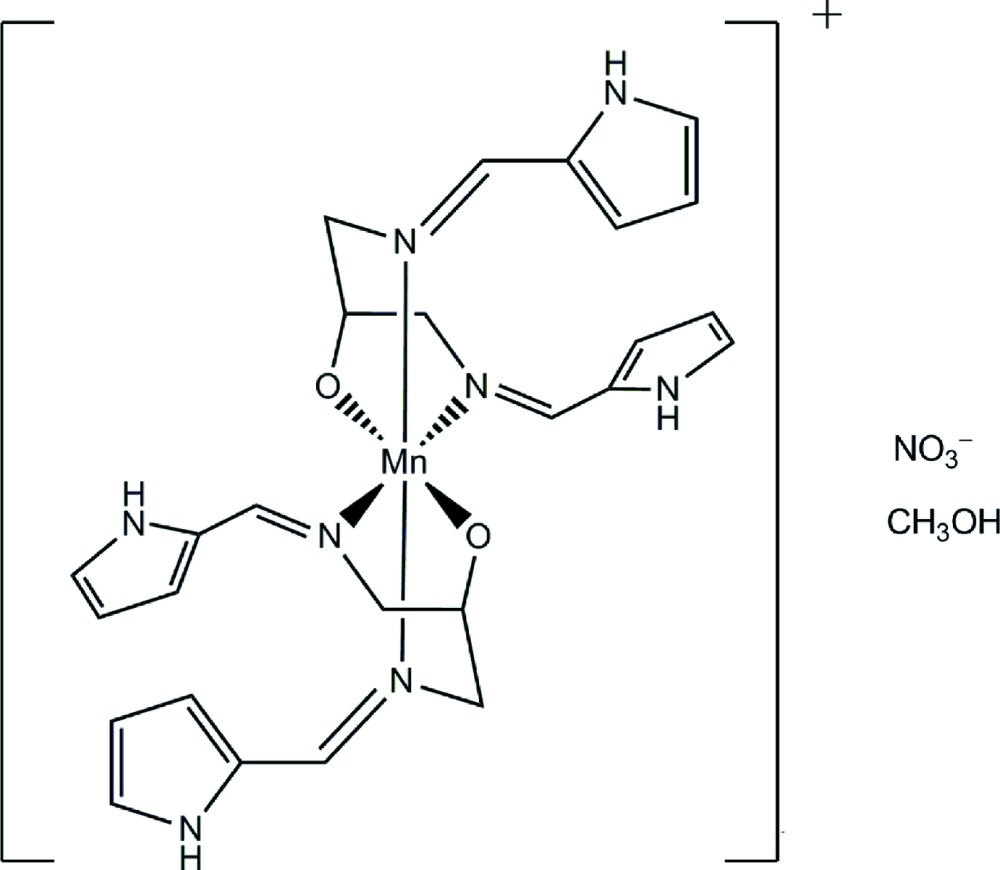


## Structural commentary   

**Figure 1:**
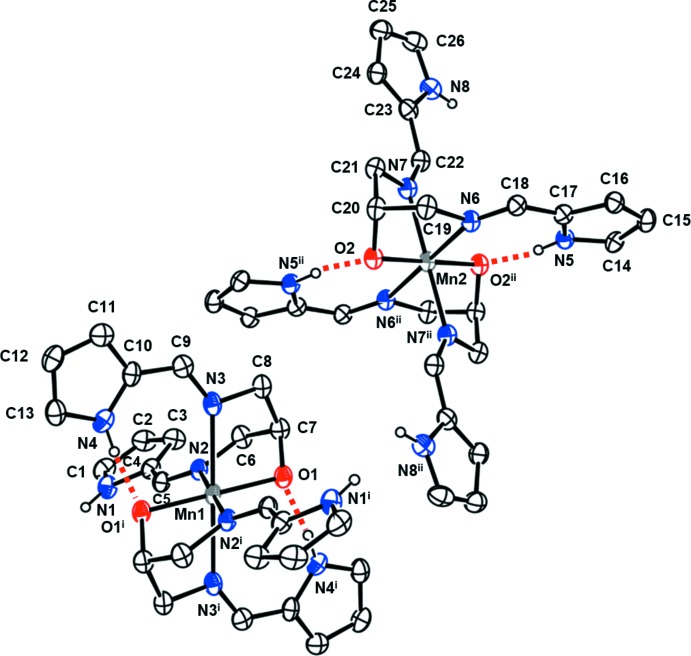
The structure of the two independent Mn^III^ complex cations in the title compound, showing the atom-labelling scheme. Displacement ellipsoids are drawn at the 30% probability level. H atoms bonded to C atoms have been omitted for clarity. Intra­molecular N—H⋯O hydrogen bonds are shown as red dashed lines. [Symmetry codes: (i) −*x* + 1, −*y* + 1, −*z* + 1; (ii) −*x* + 2, −*y* + 1, −*z*.]

The title compound crystallizes with two crystallographically independent complex mol­ecules in the asymmetric unit (Fig. 1 ▶). Each Mn^III^ ion is located on an inversion centre and is six-coordinated in a distorted octa­hedral geometry. Two bpmap ligands are coordinated to the Mn^III^ ion in a tridentate and *fac*-type manner (Berends *et al.*, 2012 ▶). That is, one O atom and one imine N of each bpmap ligand occupy in the equatorial plane and the other imine N atom is in the axial position. The pyrrole groups of both ligands are non-coordin­ating. Inter­estingly, the geometry of pyrrole groups, which results from different bpmap ligands, displays a *trans* conformation in the axial positions (Jeong *et al.*, 2014 ▶). The average equatorial bond lengths, Mn1—*L*_eq_ and Mn2—*L*_eq_, are 1.952 and 1.918 Å, respectively. The axial bond lengths, Mn1—N2 and Mn2—N6, are 2.318 (3) and 2.345 (3) Å, respectively. The axial bond lengths are much longer than the equatorial bond lengths, which can be attributed to a rather large Jahn–Teller distortion of the Mn^III^ ion (Halcrow, 2013 ▶). The bite distance (O1⋯N2) and the bite angle (N2—Mn1—O1) of the five-membered chelate ring are 2.590 (4) Å and 83.07 (10)°, respectively, while O2⋯N6 and O2—Mn2—N6 are 2.715 (3) Å and 79.26 (9)°. There are intra­molecular N—H⋯O hydrogen bonds between the pyrrole groups and the O atoms of the bpmap ligands (Fig. 1 ▶ and Table 1 ▶).

## Supra­molecular features   

**Figure 2:**
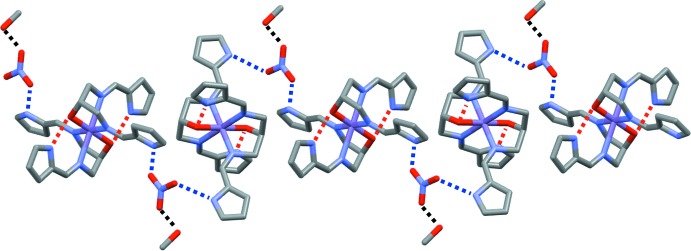
A view of the crystal packing structure of the title compound, with N—H⋯O hydrogen bonds drawn as red (intra­molecular) and blue (inter­molecular) dashed lines, and O—H⋯O hydrogen bonds drawn as black dashed lines.

The packing in the structure involves N—H⋯O hydrogen bonds between the pyrrole groups and the non-coordinating nitrate anions (Table 1 ▶), giving chains along [10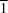]. The hy­droxy group of methanol and the nitrate ion are also connected by an O—H⋯O hydrogen bond (Fig. 2 ▶).

## Database survey   

A search of the Cambridge Structural Database (Version 5.35, November 2013 with 3 updates; Allen, 2002 ▶) indicates that only one Cu^II^ complex with the bpmap ligand has been reported (Borer & Sinn, 1998 ▶). This paper elucidates the synthesis of various pyrrole, imidazole, and salicyl­aldehyde derivatives and investigates the magnetic properties and chelating effects of Cu complexes.

### Synthesis and crystallization   

The bpmap ligand was prepared by a slight modification of the reported method (Borer & Sinn, 1998 ▶). 1,3-Di­amino­propan-2-ol (1.50 g, 0.0166 mol) was dissolved in MeOH (40 mL) followed by the addition of pyrrole-2-carbaldehyde (3.17 g, 0.0333 mol). The resulting mixture was stirred overnight at room temperature. The solvent was evaporated and the residue was dissolved in CHCl_3_. The solution was washed by concentrated brine and dried with MgSO_4_. After evaporation of the solvents under reduced pressure, an orange powder was obtained and used for the preparation of the title compound without further purification (yield: 2.98 g, 73%). ^1^H NMR (400 MHz, DMSO-*d*_6_, 293 K): δ 3.40–3.44 (*m*, 4H), 3.65 (*ddd*, *J* = 0.8, 5.1, 11.7 Hz, 2H, pyr-NH), 3.87–3.93 (*m*, 1H), 6.10 (*dd*, *J* = 3.6, 6.4 Hz, 1H, pyr), 6.44 (*dd*, *J* = 1.52, 3.4 Hz, 1H, pyr), 6.87 (*t*, *J* = 1.8 Hz, 1H, pyr), 8.05 (*s*, 2H), 11.32 (*s*, 1H, –OH). The title compound was prepared as follows: to an MeOH solution (3 mL) of Mn(NO_3_)_2_·4H_2_O (102 mg, 0.406 mmol) was added dropwise an MeOH solution (3 mL) of bpmap (50 mg, 0.205 mmol). The colour became dark orange, and then the solution was stirred for 30 min at room temperature. Black crystals of the title compound were obtained by diffusion of diethyl ether into the dark-orange solution for several days, and were collected by filtration and washed with diethyl ether and dried in air (yield: 80 mg, 33%). IR (ATR, cm^−1^): 3341, 2948, 1614, 1385, 1306.

## Refinement   

Crystal data, data collection and structure refinement details are summarized in Table 2 ▶. All H atoms were placed in geometrically idealized positions and constrained to ride on their parent atoms, with C—H distances of 0.95 (ring H atoms) and 0.95–0.99 Å (open-chain H atoms), N—H distances of 0.88 Å (ring H atoms) and O—H distances of 0.84 Å, and with *U*_iso_(H) values of 1.2 or 1.5*U*_eq_ of the parent atoms.

## Supplementary Material

Crystal structure: contains datablock(s) I. DOI: 10.1107/S1600536814020406/is5374sup1.cif

Structure factors: contains datablock(s) I. DOI: 10.1107/S1600536814020406/is5374Isup2.hkl

CCDC reference: 1023763

Additional supporting information:  crystallographic information; 3D view; checkCIF report

## Figures and Tables

**Table 1:** Hydrogen-bond geometry (Å, °)

*D*—H⋯*A*	*D*—H	H⋯*A*	*D*⋯*A*	*D*—H⋯*A*
N1—H1A⋯O4	0.88	1.96	2.800 (5)	160
N8—H8⋯O5	0.88	2.27	3.025 (5)	144
N4—H4⋯O1^i^	0.88	1.87	2.743 (4)	174
N5—H5A⋯O2^ii^	0.88	1.85	2.723 (3)	172
O6—H6⋯O3^iii^	0.84	2.05	2.781 (6)	145

Symmetry codes: (i) 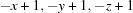; (ii) 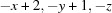; (iii) 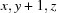.

**Table 2:** Experimental details

Crystal data	
Chemical formula	[Mn(C_13_H_15_N_4_O)_2_]NO_3_·CH_4_O
*M*_r_	635.57
Crystal system, space group	Triclinic, *P* 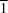
Temperature (K)	100
*a*, *b*, *c* (Å)	10.516 (2), 10.887 (2), 14.981 (3)
α, β, γ (°)	76.05 (3), 82.51 (3), 61.22 (3)
*V* (Å^3^)	1458.7 (7)
*Z*	2
Radiation type	Synchrotron, λ = 0.62998 Å
μ (mm^−1^)	0.37
Crystal size (mm)	0.08 × 0.02 × 0.02

Data collection	
Diffractometer	ADSC Q210 CCD area detector
Absorption correction	Empirical (using intensity measurements) (*HKL-3000* *SCALEPACK*; Otwinowski & Minor, 1997 ▶)
*T*_min_, *T*_max_	0.971, 0.993
No. of measured, independent and observed [*I* > 2σ(*I*)] reflections	15140, 7679, 4716
*R*_int_	0.037
(sin θ/λ)_max_ (Å^−1^)	0.696

Refinement	
*R*[*F* ^2^ > 2σ(*F* ^2^)], *wR*(*F* ^2^), *S*	0.068, 0.223, 1.04
No. of reflections	7679
No. of parameters	394
H-atom treatment	H-atom parameters constrained
Δρ_max_, Δρ_min_ (e Å^−3^)	1.35, −0.69

Computer programs: *PAL ADSC Quantum-210 ADX Program* (Arvai & Nielsen, 1983 ▶), *HKL3000sm* (Otwinowski & Minor, 1997 ▶), *SHELXS2013/1* and *SHELXL2014/6* (Sheldrick, 2008 ▶), *ORTEP-3 for Windows* and *WinGX* (Farrugia, 2012 ▶).

## supplementary crystallographic information

### Crystal data

**Table:**

[Mn(C_13_H_15_N_4_O)_2_]NO_3_·CH_4_O	*Z* = 2
*M**_r_* = 635.57	*F*(000) = 664
Triclinic, *P*1	*D*_x_ = 1.447 Mg m^−^^3^
*a* = 10.516 (2) Å	Synchrotron radiation, λ = 0.62998 Å
*b* = 10.887 (2) Å	Cell parameters from 36688 reflections
*c* = 14.981 (3) Å	θ = 0.4–33.6°
α = 76.05 (3)°	µ = 0.37 mm^−^^1^
β = 82.51 (3)°	*T* = 100 K
γ = 61.22 (3)°	Needle, black
*V* = 1458.7 (7) Å^3^	0.08 × 0.02 × 0.02 mm

### Data collection

**Table:**

ADSC Q210 CCD area detector diffractometer	4716 reflections with *I* > 2σ(*I*)
Radiation source: PLSII 2D bending magnet	*R*_int_ = 0.037
ω scan	θ_max_ = 26.0°, θ_min_ = 2.0°
Absorption correction: empirical (using intensity measurements) (*HKL-3000**SCALEPACK*; Otwinowski & Minor, 1997)	*h* = −14→14
*T*_min_ = 0.971, *T*_max_ = 0.993	*k* = −14→14
15140 measured reflections	*l* = −20→20
7679 independent reflections	

### Refinement

**Table:**

Refinement on *F*^2^	Hydrogen site location: inferred from neighbouring sites
Least-squares matrix: full	H-atom parameters constrained
*R*[*F*^2^ > 2σ(*F*^2^)] = 0.068	*w* = 1/[σ^2^(*F*_o_^2^) + (0.1401*P*)^2^] where *P* = (*F*_o_^2^ + 2*F*_c_^2^)/3
*wR*(*F*^2^) = 0.223	(Δ/σ)_max_ < 0.001
*S* = 1.04	Δρ_max_ = 1.35 e Å^−^^3^
7679 reflections	Δρ_min_ = −0.69 e Å^−^^3^
394 parameters	Extinction correction: *SHELXL*, Fc^*^=kFc[1+0.001xFc^2^λ^3^/sin(2θ)]^-1/4^
0 restraints	Extinction coefficient: 0.023 (6)

### Special details

**Table:**

Geometry. All e.s.d.'s (except the e.s.d. in the dihedral angle between two l.s. planes) are estimated using the full covariance matrix. The cell e.s.d.'s are taken into account individually in the estimation of e.s.d.'s in distances, angles and torsion angles; correlations between e.s.d.'s in cell parameters are only used when they are defined by crystal symmetry. An approximate (isotropic) treatment of cell e.s.d.'s is used for estimating e.s.d.'s involving l.s. planes.

### Fractional atomic coordinates and isotropic or equivalent isotropic displacement parameters (Å^2^)

**Table:**

	*x*	*y*	*z*	*U*_iso_*/*U*_eq_	
Mn1	0.5000	0.5000	0.5000	0.0337 (2)	
Mn2	1.0000	0.5000	0.0000	0.0370 (2)	
N1	0.3876 (3)	0.1435 (3)	0.3766 (2)	0.0470 (7)	
H1A	0.4305	0.1641	0.3249	0.056*	
N2	0.4163 (3)	0.3654 (3)	0.51136 (17)	0.0374 (6)	
N3	0.2623 (3)	0.6564 (3)	0.53278 (18)	0.0425 (6)	
N4	0.2438 (3)	0.7984 (3)	0.3331 (2)	0.0432 (6)	
H4	0.3286	0.7212	0.3413	0.052*	
N5	1.0374 (3)	0.5081 (3)	0.23664 (18)	0.0399 (6)	
H5A	1.0444	0.4558	0.1979	0.048*	
N6	0.9404 (3)	0.7068 (3)	0.05388 (18)	0.0392 (6)	
N7	0.7813 (3)	0.5863 (3)	0.00379 (19)	0.0403 (6)	
N8	0.4871 (3)	0.5318 (3)	0.1303 (2)	0.0497 (7)	
H8	0.5362	0.4546	0.1719	0.060*	
O1	0.4992 (3)	0.4489 (3)	0.63029 (15)	0.0420 (5)	
O2	0.9660 (2)	0.6340 (2)	−0.11102 (15)	0.0406 (5)	
C1	0.3287 (4)	0.0528 (4)	0.3925 (3)	0.0530 (9)	
H1	0.3268	0.0021	0.3497	0.064*	
C2	0.2728 (4)	0.0467 (4)	0.4801 (3)	0.0543 (9)	
H2	0.2259	−0.0090	0.5088	0.065*	
C3	0.2972 (4)	0.1374 (4)	0.5201 (3)	0.0501 (8)	
H3	0.2696	0.1545	0.5807	0.060*	
C4	0.3697 (4)	0.1984 (3)	0.4542 (2)	0.0411 (7)	
C5	0.4203 (3)	0.2987 (3)	0.4509 (2)	0.0394 (7)	
H5	0.4647	0.3200	0.3940	0.047*	
C6	0.3538 (4)	0.3449 (4)	0.6050 (2)	0.0476 (8)	
H6A	0.4084	0.2443	0.6380	0.057*	
H6B	0.2511	0.3679	0.6014	0.057*	
C7	0.3654 (4)	0.4475 (4)	0.6559 (2)	0.0439 (7)	
H7	0.3629	0.4117	0.7238	0.053*	
C8	0.2444 (4)	0.5988 (4)	0.6308 (2)	0.0460 (8)	
H8A	0.1492	0.5994	0.6413	0.055*	
H8B	0.2483	0.6589	0.6695	0.055*	
C9	0.1474 (4)	0.7678 (4)	0.4932 (2)	0.0448 (8)	
H9	0.0616	0.8030	0.5298	0.054*	
C10	0.1401 (4)	0.8415 (4)	0.3988 (2)	0.0418 (7)	
C11	0.0259 (4)	0.9673 (4)	0.3590 (3)	0.0494 (8)	
H11	−0.0618	1.0217	0.3894	0.059*	
C12	0.0602 (4)	1.0018 (4)	0.2666 (3)	0.0514 (9)	
H12	0.0019	1.0833	0.2222	0.062*	
C13	0.1962 (4)	0.8930 (4)	0.2529 (2)	0.0506 (9)	
H13	0.2483	0.8859	0.1959	0.061*	
C14	1.0783 (4)	0.4581 (4)	0.3259 (2)	0.0440 (8)	
H14	1.1192	0.3605	0.3567	0.053*	
C15	1.0514 (4)	0.5702 (4)	0.3644 (2)	0.0478 (8)	
H15	1.0698	0.5646	0.4260	0.057*	
C16	0.9909 (4)	0.6960 (4)	0.2954 (2)	0.0436 (7)	
H16	0.9614	0.7908	0.3020	0.052*	
C17	0.9829 (3)	0.6557 (3)	0.2167 (2)	0.0391 (7)	
C18	0.9344 (3)	0.7474 (3)	0.1285 (2)	0.0390 (7)	
H18	0.8941	0.8474	0.1251	0.047*	
C19	0.8833 (4)	0.8196 (3)	−0.0296 (2)	0.0448 (8)	
H19A	0.9579	0.8485	−0.0578	0.054*	
H19B	0.7971	0.9048	−0.0137	0.054*	
C20	0.8422 (4)	0.7605 (3)	−0.0969 (2)	0.0431 (7)	
H20	0.8145	0.8315	−0.1567	0.052*	
C21	0.7186 (4)	0.7242 (3)	−0.0626 (2)	0.0426 (7)	
H21A	0.6417	0.7999	−0.0324	0.051*	
H21B	0.6757	0.7162	−0.1146	0.051*	
C22	0.7018 (4)	0.5366 (3)	0.0565 (2)	0.0413 (7)	
H22	0.7532	0.4472	0.0971	0.050*	
C23	0.5464 (4)	0.5981 (4)	0.0621 (2)	0.0435 (7)	
C24	0.4318 (4)	0.7159 (4)	0.0125 (3)	0.0488 (8)	
H24	0.4395	0.7834	−0.0386	0.059*	
C25	0.3023 (4)	0.7167 (4)	0.0518 (3)	0.0542 (9)	
H25	0.2065	0.7835	0.0318	0.065*	
C26	0.3408 (4)	0.6038 (5)	0.1238 (3)	0.0587 (10)	
H26	0.2751	0.5789	0.1637	0.070*	
N9	0.6664 (4)	0.1756 (3)	0.2130 (2)	0.0536 (8)	
O3	0.7146 (4)	0.0487 (3)	0.2126 (3)	0.0847 (10)	
O4	0.5335 (4)	0.2489 (3)	0.2351 (2)	0.0736 (9)	
O5	0.7396 (4)	0.2393 (3)	0.1937 (2)	0.0816 (10)	
C27	0.5483 (5)	0.8915 (5)	0.1434 (3)	0.0647 (11)	
H27A	0.5612	0.9497	0.0855	0.097*	
H27B	0.4776	0.8609	0.1351	0.097*	
H27C	0.6414	0.8070	0.1616	0.097*	
O6	0.4984 (4)	0.9716 (4)	0.2111 (2)	0.0767 (9)	
H6	0.5689	0.9692	0.2335	0.115*	

### Atomic displacement parameters (Å^2^)

**Table:**

	*U*^11^	*U*^22^	*U*^33^	*U*^12^	*U*^13^	*U*^23^
Mn1	0.0435 (4)	0.0353 (4)	0.0310 (3)	−0.0242 (3)	−0.0070 (3)	−0.0055 (2)
Mn2	0.0459 (4)	0.0355 (4)	0.0377 (4)	−0.0254 (3)	−0.0034 (3)	−0.0060 (3)
N1	0.0617 (18)	0.0434 (16)	0.0475 (16)	−0.0297 (14)	−0.0076 (13)	−0.0146 (12)
N2	0.0460 (15)	0.0404 (14)	0.0355 (13)	−0.0270 (12)	−0.0055 (11)	−0.0069 (10)
N3	0.0583 (17)	0.0484 (16)	0.0352 (14)	−0.0351 (14)	−0.0027 (12)	−0.0098 (11)
N4	0.0473 (15)	0.0349 (14)	0.0526 (16)	−0.0219 (12)	−0.0139 (13)	−0.0054 (11)
N5	0.0443 (15)	0.0419 (15)	0.0417 (14)	−0.0248 (12)	−0.0028 (11)	−0.0116 (11)
N6	0.0475 (15)	0.0378 (14)	0.0406 (14)	−0.0263 (12)	−0.0022 (11)	−0.0074 (11)
N7	0.0485 (15)	0.0392 (14)	0.0425 (14)	−0.0268 (12)	−0.0022 (12)	−0.0099 (11)
N8	0.0544 (18)	0.0510 (18)	0.0534 (17)	−0.0321 (15)	0.0050 (14)	−0.0145 (14)
O1	0.0514 (13)	0.0506 (13)	0.0365 (11)	−0.0325 (11)	−0.0047 (10)	−0.0092 (9)
O2	0.0518 (13)	0.0394 (12)	0.0390 (11)	−0.0280 (11)	−0.0031 (10)	−0.0068 (9)
C1	0.056 (2)	0.046 (2)	0.070 (2)	−0.0280 (17)	−0.0115 (18)	−0.0204 (17)
C2	0.054 (2)	0.051 (2)	0.076 (3)	−0.0359 (18)	0.0054 (18)	−0.0225 (18)
C3	0.053 (2)	0.047 (2)	0.063 (2)	−0.0301 (17)	0.0031 (17)	−0.0195 (16)
C4	0.0455 (17)	0.0372 (16)	0.0471 (18)	−0.0219 (14)	−0.0083 (14)	−0.0103 (13)
C5	0.0455 (17)	0.0408 (17)	0.0385 (16)	−0.0242 (14)	−0.0054 (13)	−0.0082 (13)
C6	0.061 (2)	0.051 (2)	0.0452 (18)	−0.0369 (18)	0.0021 (16)	−0.0125 (15)
C7	0.0526 (19)	0.0461 (18)	0.0409 (17)	−0.0294 (16)	−0.0019 (14)	−0.0075 (14)
C8	0.058 (2)	0.051 (2)	0.0369 (17)	−0.0315 (17)	−0.0039 (14)	−0.0056 (14)
C9	0.052 (2)	0.0451 (19)	0.0491 (19)	−0.0303 (16)	−0.0017 (15)	−0.0136 (15)
C10	0.058 (2)	0.0419 (17)	0.0392 (16)	−0.0331 (16)	−0.0110 (14)	−0.0049 (13)
C11	0.0506 (19)	0.048 (2)	0.060 (2)	−0.0288 (17)	−0.0009 (16)	−0.0181 (16)
C12	0.062 (2)	0.0414 (19)	0.054 (2)	−0.0277 (17)	−0.0198 (17)	0.0022 (15)
C13	0.066 (2)	0.059 (2)	0.0411 (18)	−0.042 (2)	−0.0040 (16)	−0.0065 (15)
C14	0.0443 (18)	0.0463 (19)	0.0465 (18)	−0.0240 (15)	−0.0103 (14)	−0.0067 (14)
C15	0.055 (2)	0.057 (2)	0.0446 (18)	−0.0347 (18)	−0.0076 (15)	−0.0107 (15)
C16	0.0478 (18)	0.0464 (19)	0.0471 (18)	−0.0272 (15)	−0.0020 (14)	−0.0158 (14)
C17	0.0416 (16)	0.0408 (17)	0.0445 (17)	−0.0262 (14)	−0.0005 (13)	−0.0102 (13)
C18	0.0435 (17)	0.0384 (16)	0.0450 (17)	−0.0249 (14)	−0.0028 (13)	−0.0118 (13)
C19	0.061 (2)	0.0343 (16)	0.0458 (18)	−0.0278 (16)	−0.0096 (15)	−0.0033 (13)
C20	0.057 (2)	0.0350 (16)	0.0403 (17)	−0.0245 (15)	−0.0095 (15)	−0.0026 (13)
C21	0.0502 (19)	0.0370 (17)	0.0445 (18)	−0.0231 (15)	−0.0075 (14)	−0.0052 (13)
C22	0.0516 (19)	0.0383 (17)	0.0437 (17)	−0.0270 (15)	−0.0043 (14)	−0.0102 (13)
C23	0.0526 (19)	0.0488 (19)	0.0453 (18)	−0.0334 (16)	0.0038 (14)	−0.0186 (14)
C24	0.053 (2)	0.053 (2)	0.0489 (19)	−0.0288 (17)	−0.0054 (16)	−0.0147 (15)
C25	0.047 (2)	0.060 (2)	0.065 (2)	−0.0272 (18)	0.0007 (17)	−0.0254 (19)
C26	0.052 (2)	0.064 (3)	0.077 (3)	−0.036 (2)	0.0161 (19)	−0.033 (2)
N9	0.060 (2)	0.0422 (17)	0.062 (2)	−0.0288 (16)	−0.0171 (16)	0.0033 (14)
O3	0.091 (2)	0.0600 (19)	0.115 (3)	−0.0362 (18)	−0.017 (2)	−0.0284 (19)
O4	0.106 (3)	0.0543 (17)	0.0586 (18)	−0.0387 (18)	0.0115 (17)	−0.0116 (13)
O5	0.091 (2)	0.0552 (18)	0.104 (3)	−0.0405 (18)	−0.040 (2)	0.0090 (16)
C27	0.061 (2)	0.077 (3)	0.067 (3)	−0.032 (2)	0.005 (2)	−0.038 (2)
O6	0.086 (2)	0.082 (2)	0.075 (2)	−0.045 (2)	0.0010 (18)	−0.0264 (17)

### Geometric parameters (Å, º)

**Table:**

Mn1—O1	1.896 (2)	C8—H8A	0.9900
Mn1—N2	2.008 (2)	C8—H8B	0.9900
Mn1—N3	2.318 (3)	C9—C10	1.440 (5)
Mn2—O2	1.872 (2)	C9—H9	0.9500
Mn2—N7	2.021 (3)	C10—C11	1.372 (5)
Mn2—N6	2.345 (3)	C11—C12	1.391 (5)
N1—C1	1.361 (4)	C11—H11	0.9500
N1—C4	1.383 (4)	C12—C13	1.379 (6)
N1—H1A	0.8800	C12—H12	0.9500
N2—C5	1.276 (4)	C13—H13	0.9500
N2—C6	1.483 (4)	C14—C15	1.366 (5)
N3—C9	1.309 (4)	C14—H14	0.9500
N3—C8	1.477 (4)	C15—C16	1.414 (5)
N4—C13	1.348 (4)	C15—H15	0.9500
N4—C10	1.352 (5)	C16—C17	1.379 (4)
N4—H4	0.8800	C16—H16	0.9500
N5—C14	1.357 (4)	C17—C18	1.432 (4)
N5—C17	1.387 (4)	C18—H18	0.9500
N5—H5A	0.8800	C19—C20	1.520 (4)
N6—C18	1.282 (4)	C19—H19A	0.9900
N6—C19	1.476 (4)	C19—H19B	0.9900
N7—C22	1.294 (4)	C20—C21	1.528 (5)
N7—C21	1.474 (4)	C20—H20	1.0000
N8—C26	1.353 (5)	C21—H21A	0.9900
N8—C23	1.366 (4)	C21—H21B	0.9900
N8—H8	0.8800	C22—C23	1.436 (5)
O1—C7	1.416 (4)	C22—H22	0.9500
O2—C20	1.407 (4)	C23—C24	1.391 (5)
C1—C2	1.366 (6)	C24—C25	1.409 (5)
C1—H1	0.9500	C24—H24	0.9500
C2—C3	1.404 (5)	C25—C26	1.354 (6)
C2—H2	0.9500	C25—H25	0.9500
C3—C4	1.405 (5)	C26—H26	0.9500
C3—H3	0.9500	N9—O3	1.223 (4)
C4—C5	1.416 (4)	N9—O5	1.231 (4)
C5—H5	0.9500	N9—O4	1.278 (4)
C6—C7	1.553 (4)	C27—O6	1.382 (5)
C6—H6A	0.9900	C27—H27A	0.9800
C6—H6B	0.9900	C27—H27B	0.9800
C7—C8	1.511 (5)	C27—H27C	0.9800
C7—H7	1.0000	O6—H6	0.8400
			
O1^i^—Mn1—O1	180.0	C7—C8—H8B	110.0
O1^i^—Mn1—N2	96.93 (10)	H8A—C8—H8B	108.4
O1—Mn1—N2	83.07 (10)	N3—C9—C10	125.5 (3)
N2—Mn1—N2^i^	180.0	N3—C9—H9	117.2
O1^i^—Mn1—N3	100.41 (10)	C10—C9—H9	117.2
O1—Mn1—N3	79.59 (10)	N4—C10—C11	107.7 (3)
N2—Mn1—N3	82.41 (10)	N4—C10—C9	126.2 (3)
N2^i^—Mn1—N3	97.59 (10)	C11—C10—C9	126.1 (3)
N3—Mn1—N3^i^	180.0	C10—C11—C12	108.5 (3)
O2^ii^—Mn2—O2	180.0	C10—C11—H11	125.8
O2^ii^—Mn2—N7	96.98 (11)	C12—C11—H11	125.8
O2—Mn2—N7	83.02 (11)	C13—C12—C11	105.6 (3)
N7—Mn2—N7^ii^	180.0	C13—C12—H12	127.2
O2^ii^—Mn2—N6	100.74 (9)	C11—C12—H12	127.2
O2—Mn2—N6	79.26 (9)	N4—C13—C12	109.2 (3)
N7—Mn2—N6	80.35 (10)	N4—C13—H13	125.4
N7^ii^—Mn2—N6	99.65 (10)	C12—C13—H13	125.4
N6—Mn2—N6^ii^	180.0	N5—C14—C15	109.3 (3)
C1—N1—C4	109.4 (3)	N5—C14—H14	125.4
C1—N1—H1A	125.3	C15—C14—H14	125.4
C4—N1—H1A	125.3	C14—C15—C16	107.2 (3)
C5—N2—C6	122.7 (3)	C14—C15—H15	126.4
C5—N2—Mn1	127.1 (2)	C16—C15—H15	126.4
C6—N2—Mn1	110.10 (19)	C17—C16—C15	107.2 (3)
C9—N3—C8	116.0 (3)	C17—C16—H16	126.4
C9—N3—Mn1	141.1 (2)	C15—C16—H16	126.4
C8—N3—Mn1	102.9 (2)	C16—C17—N5	107.7 (3)
C13—N4—C10	109.0 (3)	C16—C17—C18	126.6 (3)
C13—N4—H4	125.5	N5—C17—C18	125.6 (3)
C10—N4—H4	125.5	N6—C18—C17	125.9 (3)
C14—N5—C17	108.5 (3)	N6—C18—H18	117.0
C14—N5—H5A	125.7	C17—C18—H18	117.0
C17—N5—H5A	125.7	N6—C19—C20	108.3 (3)
C18—N6—C19	117.2 (3)	N6—C19—H19A	110.0
C18—N6—Mn2	140.8 (2)	C20—C19—H19A	110.0
C19—N6—Mn2	101.82 (18)	N6—C19—H19B	110.0
C22—N7—C21	122.4 (3)	C20—C19—H19B	110.0
C22—N7—Mn2	128.0 (2)	H19A—C19—H19B	108.4
C21—N7—Mn2	109.6 (2)	O2—C20—C19	107.1 (3)
C26—N8—C23	109.2 (3)	O2—C20—C21	108.2 (2)
C26—N8—H8	125.4	C19—C20—C21	113.9 (3)
C23—N8—H8	125.4	O2—C20—H20	109.2
C7—O1—Mn1	105.75 (18)	C19—C20—H20	109.2
C20—O2—Mn2	106.92 (19)	C21—C20—H20	109.2
N1—C1—C2	108.9 (3)	N7—C21—C20	107.0 (3)
N1—C1—H1	125.5	N7—C21—H21A	110.3
C2—C1—H1	125.5	C20—C21—H21A	110.3
C1—C2—C3	107.7 (3)	N7—C21—H21B	110.3
C1—C2—H2	126.2	C20—C21—H21B	110.3
C3—C2—H2	126.2	H21A—C21—H21B	108.6
C2—C3—C4	107.4 (3)	N7—C22—C23	128.8 (3)
C2—C3—H3	126.3	N7—C22—H22	115.6
C4—C3—H3	126.3	C23—C22—H22	115.6
N1—C4—C3	106.5 (3)	N8—C23—C24	106.9 (3)
N1—C4—C5	118.3 (3)	N8—C23—C22	117.8 (3)
C3—C4—C5	135.1 (3)	C24—C23—C22	135.3 (3)
N2—C5—C4	130.9 (3)	C23—C24—C25	107.5 (4)
N2—C5—H5	114.6	C23—C24—H24	126.3
C4—C5—H5	114.6	C25—C24—H24	126.3
N2—C6—C7	106.8 (3)	C26—C25—C24	106.7 (4)
N2—C6—H6A	110.4	C26—C25—H25	126.6
C7—C6—H6A	110.4	C24—C25—H25	126.6
N2—C6—H6B	110.4	N8—C26—C25	109.7 (4)
C7—C6—H6B	110.4	N8—C26—H26	125.2
H6A—C6—H6B	108.6	C25—C26—H26	125.2
O1—C7—C8	108.4 (3)	O3—N9—O5	123.6 (4)
O1—C7—C6	108.2 (3)	O3—N9—O4	119.8 (3)
C8—C7—C6	112.0 (3)	O5—N9—O4	116.6 (3)
O1—C7—H7	109.4	O6—C27—H27A	109.5
C8—C7—H7	109.4	O6—C27—H27B	109.5
C6—C7—H7	109.4	H27A—C27—H27B	109.5
N3—C8—C7	108.4 (3)	O6—C27—H27C	109.5
N3—C8—H8A	110.0	H27A—C27—H27C	109.5
C7—C8—H8A	110.0	H27B—C27—H27C	109.5
N3—C8—H8B	110.0	C27—O6—H6	109.5
			
N2—Mn1—O1—C7	−41.9 (2)	C9—C10—C11—C12	−179.0 (3)
N2^i^—Mn1—O1—C7	138.1 (2)	C10—C11—C12—C13	0.5 (4)
N3—Mn1—O1—C7	41.56 (19)	C10—N4—C13—C12	1.3 (4)
N3^i^—Mn1—O1—C7	−138.44 (19)	C11—C12—C13—N4	−1.1 (4)
N7—Mn2—O2—C20	39.63 (19)	C17—N5—C14—C15	0.0 (4)
N7^ii^—Mn2—O2—C20	−140.37 (19)	N5—C14—C15—C16	−0.1 (4)
N6—Mn2—O2—C20	−41.82 (18)	C14—C15—C16—C17	0.1 (4)
N6^ii^—Mn2—O2—C20	138.18 (18)	C15—C16—C17—N5	−0.1 (4)
C4—N1—C1—C2	−0.3 (4)	C15—C16—C17—C18	−177.1 (3)
N1—C1—C2—C3	0.3 (4)	C14—N5—C17—C16	0.1 (4)
C1—C2—C3—C4	−0.1 (4)	C14—N5—C17—C18	177.1 (3)
C1—N1—C4—C3	0.3 (4)	C19—N6—C18—C17	179.6 (3)
C1—N1—C4—C5	−178.3 (3)	Mn2—N6—C18—C17	6.1 (6)
C2—C3—C4—N1	−0.1 (4)	C16—C17—C18—N6	172.9 (3)
C2—C3—C4—C5	178.1 (4)	N5—C17—C18—N6	−3.6 (5)
C6—N2—C5—C4	1.1 (6)	C18—N6—C19—C20	−156.8 (3)
Mn1—N2—C5—C4	177.2 (3)	Mn2—N6—C19—C20	19.0 (3)
N1—C4—C5—N2	179.3 (3)	Mn2—O2—C20—C19	67.7 (3)
C3—C4—C5—N2	1.4 (7)	Mn2—O2—C20—C21	−55.4 (3)
C5—N2—C6—C7	−178.4 (3)	N6—C19—C20—O2	−55.2 (4)
Mn1—N2—C6—C7	5.0 (3)	N6—C19—C20—C21	64.3 (4)
Mn1—O1—C7—C8	−66.4 (3)	C22—N7—C21—C20	168.6 (3)
Mn1—O1—C7—C6	55.3 (3)	Mn2—N7—C21—C20	−9.4 (3)
N2—C6—C7—O1	−38.4 (4)	O2—C20—C21—N7	41.0 (3)
N2—C6—C7—C8	81.0 (3)	C19—C20—C21—N7	−77.9 (3)
C9—N3—C8—C7	161.2 (3)	C21—N7—C22—C23	0.4 (5)
Mn1—N3—C8—C7	−16.6 (3)	Mn2—N7—C22—C23	178.1 (2)
O1—C7—C8—N3	53.1 (3)	C26—N8—C23—C24	0.5 (4)
C6—C7—C8—N3	−66.2 (3)	C26—N8—C23—C22	−179.2 (3)
C8—N3—C9—C10	179.7 (3)	N7—C22—C23—N8	−173.4 (3)
Mn1—N3—C9—C10	−3.6 (6)	N7—C22—C23—C24	7.0 (6)
C13—N4—C10—C11	−1.0 (4)	N8—C23—C24—C25	−1.1 (4)
C13—N4—C10—C9	178.3 (3)	C22—C23—C24—C25	178.6 (3)
N3—C9—C10—N4	8.5 (5)	C23—C24—C25—C26	1.2 (4)
N3—C9—C10—C11	−172.3 (3)	C23—N8—C26—C25	0.3 (4)
N4—C10—C11—C12	0.3 (4)	C24—C25—C26—N8	−0.9 (4)

Symmetry codes: (i) −*x*+1, −*y*+1, −*z*+1; (ii) −*x*+2, −*y*+1, −*z*.

### Hydrogen-bond geometry (Å, º)

**Table:**

*D*—H···*A*	*D*—H	H···*A*	*D*···*A*	*D*—H···*A*
N1—H1*A*···O4	0.88	1.96	2.800 (5)	160
N8—H8···O5	0.88	2.27	3.025 (5)	144
N4—H4···O1^i^	0.88	1.87	2.743 (4)	174
N5—H5*A*···O2^ii^	0.88	1.85	2.723 (3)	172
O6—H6···O3^iii^	0.84	2.05	2.781 (6)	145

Symmetry codes: (i) −*x*+1, −*y*+1, −*z*+1; (ii) −*x*+2, −*y*+1, −*z*; (iii) *x*, *y*+1, *z*.
